# The Application of *Trichoderma* Strains or Metabolites Alters the Olive Leaf Metabolome and the Expression of Defense-Related Genes

**DOI:** 10.3390/jof6040369

**Published:** 2020-12-16

**Authors:** Roberta Marra, Mariangela Coppola, Angela Pironti, Filomena Grasso, Nadia Lombardi, Giada d’Errico, Andrea Sicari, Sergio Bolletti Censi, Sheridan L. Woo, Rosa Rao, Francesco Vinale

**Affiliations:** 1 Department of Agricultural Sciences, University of Naples Federico II, Portici, 80055 Naples, Italy; mariangela.coppola@unina.it (M.C.); angela.pironti@unina.it (A.P.); filomena.grasso@unina.it (F.G.); nadia.lombardi@unina.it (N.L.); giada.derrico@unina.it (G.d.); rao@unina.it (R.R.); 2 BAT Center-Interuniversity Center for Studies on Bioinspired Agro-Environmental Technology, University of Naples Federico II, Portici, 80055 Naples, Italy; woo@unina.it (S.L.W.); frvinale@unina.it (F.V.); 3 Linfa S.c.a r.l., 89900 Vibo Valentia, Italy; andrea@laboratoriolinfa.it (A.S.); sergiobolletti@cosvitec.eu (S.B.C.); 4 Department of Pharmacy, University of Naples Federico II, 80131 Naples, Italy; 5 Task Force on Microbiome Studies, University of Naples Federico II, 80131 Naples, Italy; 6 Department of Veterinary Medicine and Animal Productions, University of Naples Federico II, 80137 Naples, Italy; 7 Institute for Sustainable Plant Protection, National Research Council, Portici, 80055 Naples, Italy

**Keywords:** *Olea europaea*, *Trichoderma*, secondary metabolites, defense-related genes, real-time RT-PCR, LC–MS Q-TOF, phenolic compounds, oleuropein, *Fusicladium oleagineum*

## Abstract

Biocontrol fungal strains of the genus *Trichoderma* can antagonize numerous plant pathogens and promote plant growth using different mechanisms of action, including the production of secondary metabolites (SMs). In this work we analyzed the effects of repeated applications of selected *Trichoderma* strains or SMs on young olive trees on the stimulation of plant growth and on the development of olive leaf spot disease caused by *Fusicladium oleagineum*. In addition, metabolomic analyses and gene expression profiles of olive leaves were carried out by LC–MS Q-TOF and real-time RT-PCR, respectively. A total of 104 phenolic compounds were detected from olive leave extracts and 20 were putatively identified. Targeted and untargeted approaches revealed significant differences in both the number and type of phenolic compounds accumulated in olive leaves after *Trichoderma* applications, as compared to water-treated plants. Different secoiridoids were less abundant in treated plants than in controls, while the accumulation of flavonoids (including luteolin and apigenin derivatives) increased following the application of specific *Trichoderma* strain. The induction of defense-related genes, and of genes involved in the synthesis of the secoiridoid oleuropein, was also analyzed and revealed a significant variation of gene expression according to the strain or metabolite applied.

## 1. Introduction

Olive (*Olea europaea* L.) has played a fundamental role for the development of Mediterranean civilizations in the economic, social, and cultural areas [1,2]. It is the second most important oil fruit crop in the world, after oil palm, cultivated over 8 million hectares of land, largely concentrated in the Mediterranean basin [3,4].

A large body of epidemiological studies associates olive oil consumption with a decreased frequency of cardiovascular disease and it has demonstrated cancer-protective effects [5]. In addition, olive leaves represent a valuable waste byproduct of olive cultivation and processing, rich of bioactive compounds [6,7]. These include phenolic compounds, which have shown numerous positive effects on human health (i.e., antihypertensive, antiinflammatory, hypoglycemic, antimicrobial, and hypocholesterolemic) that were mainly related to an antioxidative action (see [7] for a review). Olive tree leaf extracts are also used in antiaging cosmetics due to their pronounced antioxidant activity, and in the food industry as integrators, preservers or flavorings, thus opening multiple opportunities in the field of functional foods [8,9,10].

Biophenols in olive leaves include a large number of compounds, grouped as phenolic acids, simple phenols, secoiridoids, and flavonoids. The secoiridoid oleuropein and its derivatives are the principal components of olive leaves, and oleuropein is generally the most abundant. Several factors may influence the qualitative and quantitative phenolic profile of olive leaf, such as the date of collection [11], drying conditions [12], cultivation area [13], extraction procedure [13,14], and cultivar genetic background [14,15]. Recent studies investigated the olive leaf phenolic pattern from various cultivars or at different growth stages using liquid chromatography coupled to mass spectrometry (LC–MS) [8,12,16,17,18,19,20].

Phenolic compounds are produced by olive plants in response to pathogen attack or abiotic stress and have been associated with plant host resistance [21,22,23,24,25,26]. Previous studies reported the resistance of some olive cultivars to Verticillium wilt and olive scab as related to a multifactorial phenolic component mainly represented by tyrosol and its derivatives, oleuropein and rutin [27,28].

Microbial biocontrol agents (MBCAs) may exert multiple beneficial effects on plants, including direct pathogen control, growth promotion, and induction of disease resistance [29,30,31]. *Trichoderma* spp. are filamentous fungi marketed worldwide as the active ingredients of over 50 biofungicides able to inhibit both aerial and soil borne diseases, especially root rot caused by *Fusarium* spp., *Rhizoctonia* spp., and *Pythium* spp. [32,33]. *Trichoderma* fungi are also well known as plant growth promoters (PGPs) on several crops, including solanaceae, cucurbits, and ornamentals, and their beneficial effects include a higher development of the root system and leaf area, an increase in production yields, and the induction of plant defenses against numerous pathogens [34,35]. Recent findings highlighted the involvement of secondary metabolites (SMs) produced by these beneficial fungi in the interactions with the plant and showed their involvement in the mechanisms of action of the living microbe [36,37,38,39,40,41,42]. Carrero-Carrón et al. reported the ability of *T. asperellum* to colonize olive rhizosphere and promote plant growth and reduce the severity of disease symptoms caused by highly virulent defoliating pathotype of *Verticillium dahliae* [43].

Even though the biocontrol mechanisms employed by *Trichoderma* spp. have received considerable attention in the last decades, there is still little information about the effect on plant metabolome and gene expression of the application of MBCAs or their metabolites. In the present study, we investigated the effect of in vivo treatment of selected *Trichoderma* strains or metabolites on the growth and disease resistance of olive trees. In addition, the metabolome of the treated leaf extracts and the expression of genes involved in defense responses and in oleuropein biosynthesis were also analyzed.

## 2. Materials and Methods

### 2.1. Microbial Strains, Bioactive Metabolites, and Culture Conditions

In this work different *Trichoderma* species (*T. harzianum* strains M10, TH1, and T22; *T. virens* strain GV41 and *T. asperellum* strain KV906) were used. Fungal cultures were obtained from the collection available at the Department of Agricultural Sciences of the University of Naples Federico II and cultivated as described previously [44]. Strains T22 and GV41 are registered as the active ingredients of bioformulations used as plant protection products and approved for use by the US EPA and European Commission [45]. Spore suspensions were adjusted to the desired concentrations by a hemocytometer.

The *Trichoderma* bioactive metabolites 6-pentyl-α-pyrone (6PP) and harzianic acid (HA) were used for the treatments on olive trees. 6PP was isolated from the liquid culture of *T. atroviride* strain P1 as reported by Vinale et al. [46], whereas HA was extracted from the liquid culture of *T. harzianum* strain M10 as previously described [37]. Metabolite solutions were prepared by diluting the compound with distilled water up to the final concentration used for the treatments. For both HA and 6PP, 0.01% ethyl acetate was added to facilitate resuspension and was successively evaporated under cabinet flow.

### 2.2. Plant Material and Experimental Design

Experiments were conducted on 2 yr-old olive trees (*Olea europaea* L.) cv. Carolea, which is typically cultivated in the south of Italy. The plants were transplanted into plastic pots (50 cm diameter × 40 cm high, one plant per pot) and placed in a field at the Department of Agricultural Sciences of the University of Naples Federico II located in Portici (Naples, Italy). Each pot contained 50 L of universal soil containing peat, granulated pumice, and coconut fiber. Plants were watered once a week to field capacity. No additional nutrients were added. The experiments consisted of 8 treatments, including water control, using 5 *Trichoderma* strains (M10, TH1, KV906, GV41, or T22) and 2 metabolites (HA or 6PP). The field trial was arranged in a completely randomized block design.

Olive trees were treated with *Trichoderma* spore suspensions (1 × 10^7^ sp mL^−1^) or fungal metabolite solutions (1 × 10^−5^ M) through root system exposure, as follows: once at the time of transplant by root dip (10 min, 1 L plant^−1^), and repeated every 30 days by soil irrigation (400 mL plant^−1^), for a total of 6 applications. Overall, each treatment was applied to 15 plants (3 biological replicates per treatment and 5 plants in each replicate). Fifteen days after each application, leaf samples were collected from each plant and stored at −80 °C until use.

The number of leaves naturally infected by *Fusicladium oleagineum* (syn. *Spilocaea oleagina*) was estimated visually by an early diagnosis method [47]. The chlorophyll content of olive leaves was estimated by the average of twenty readings per sample using a chlorophyll meter (SPAD-502Plus, Konica Minolta Sensing Europe B.V., Nieuwegein, The Netherlands), as reported previously [48].

Data from bioassays were subjected to statistical analysis using SPSS V24 statistic software (IBM Corporation, Armonk, NY, USA). Significant differences among infected leaves were estimated according to the Student’s *t*-test with a 0.05 level of significance. The data of SPAD index were analyzed by one-way ANOVA and significant differences among treatments were compared using S–N–K (Student–Newman–Keuls) and Fisher’s least significant difference (LSD) post hoc tests at the 0.05 level of significance.

### 2.3. Relative Quantification of Gene Expression

Olive leaves collected from each treatment 15 days after the last application of spores/metabolites were used for plant gene expression analysis through real-time reverse transcription-polymerase chain reaction (real time RT-PCR). The isolation of total RNA from olive leaves, the synthesis of the first strand cDNA and real-time PCR were performed as previously reported [49]. Gene expression analysis was carried out using two technical replicates for each of the three biological replicates per sample. Relative quantification of gene expression was carried out using the 2^−ΔΔCt^ method [50]. The statistical significance was evaluated using the Student’s *t*-test. The housekeeping gene elongation factor 1-α (EF1α) was used as an endogenous reference gene for the normalization of the expression levels of the target genes. Primers and their main features are reported in Table S2.

### 2.4. Extraction of Phenolic Compound from Olive Leaves

Phenolic compounds were extracted from olive leaves as previously reported by Talhaoui et al. [8], with some modifications. Briefly, 200 mg of freeze-dried leaves were crushed and extracted twice with 5 mL of methanol/water (80/20, *v*/*v*) in an ultrasonic bath (10 min), and then centrifuged at 4000 rpm, 4 °C for 10 min. The supernatants were collected, dried in a speed-vac (Savant SpeedVac, Thermo Fisher Scientific, Waltham, MA, USA), and resuspended in 2 mL of methanol/water (50/50, *v*/*v*). Samples were then filtered (Millipore 0.45 μm) and stored at −20 °C in the dark until use.

### 2.5. Determination of Phenolic Compounds by LC–MS Q-TOF

The analyses of phenolic extracts from olive leaves was performed on an Agilent HP 1260 Infinity Series liquid chromatography equipped with a DAD system (Agilent Technologies, Santa Clara, CA, USA) coupled to a Q-TOF mass spectrometer model G6540B (Agilent Technologies, Santa Clara, CA, USA). Separations were performed using a Zorbax Eclips Plus C-18 column (2.1 mm × 100 mm, 2.7 μm) from Agilent Technologies, maintained at 25 °C. The analyses were performed using a linear gradient system composed of 0.1% (*v*/*v*) formic acid in water (phase A), and 0.1% (*v*/*v*) formic acid in acetonitrile (phase B). The flow was 0.5 mL min^−1^, and the solvent gradient changed according to the following conditions: 0 min, 5% B; 4 min, 9% B; 7 min, 12% B; 8 min, 15% B; 9min, 16% B; 14 min, 20% B; 15 min, 22% B; 18 min, 28% B; 19 min, 30% B; 20 min, 31% B; 21.50 min, 32% B; 23 min, 34% B; 24 min, 35% B; 25.5 min, 40% B; 27 min, 50% B; 30 min, 100% B; 35 min, 100% B; and 37 min, 5% B.

The UV spectra were collected by DAD every 0.4 s from 190 to 750 nm with a resolution of 2 nm. The MS system was equipped with a dual electrospray ionization (ESI) source and operated with Agilent MassHunter Data Acquisition Software, version B.05.01 in the negative mode as previously reported [51]. Briefly, the mass spectra were acquired in the mass range 100–1600 *m*/*z* with 3 scans per second. Two reference mass compounds were injected in the source at a constant flow of 0.060 mL min^−1^, to perform the real-time lock mass correction: purine (C_5_H_4_N_4_ at *m*/*z* 121.050873, 10 µmol L^−1^) and hexakis (1H,1H, 3H-tetrafluoropentoxy)-phosphazene (C_18_H_18_O_6_N_3_P_3_F_24_ at *m*/*z* 922.009798, 2 µmol L^−1^). The capillary was maintained at 4000 V, Fragmentor at 180 V, cone 1 (skimmer 1) at 45 V. Gas temperature was 350 °C during the run at 11 L min^−1^, and the nebulizer was set at 45 psig. The injected sample volume was 5 µL.

Solvents were of LC–MS grade, and all other chemicals were of analytical grade (from Sigma-Aldrich, Germany, unless otherwise stated; ESI–TOF tune mix from Agilent Technologies).

Three biological samples were analyzed for each treatment and each run was repeated 3 times. Data were evaluated using MassHunter Qualitative Analysis Software version B.06.00 (Agilent Technologies) and comparisons were made to known compounds in an in-house database combined with data from the existing literature. Positive identifications of plant metabolites were considered for analysis if the compound was detected with a mass error below 10 ppm and with a sufficient score.

The quantification of phenolic compounds in the olive leaf extracts was performed using four commercial standards (oleuropein, hydroxytyrosol, apigenin, and luteolin) purchased from Sigma-Aldrich. The standards were diluted in methanol: water (50:50, *v*/*v*) and injected into the system to prepare standard calibration curves (Table S3).

### 2.6. Data Analysis and Compounds Identification

All chromatographic separations were run in triplicate (technical replicate) and the extraction of phenolic compound was repeated 3 times per treatment (biological replicate). Hence, each sample consisted of 9 replicates obtained from olive leaves of plants treated with *Trichoderma* strains or metabolites, and pickled monthly for a total of 5 samplings. Values of different results were expressed as the means mg/g olive leaves. Mass Profiler Professional (Agilent Technologies, MPP v 13.1.1, Santa Clara, CA, USA) was used for molecular feature normalization and alignment, statistical analysis, and compound identification.

MPP normalization and alignment parameters were as follows: abundance filter, >5000 counts; minimum number of ions, 2; alignment RT window, 0.4 min intercept, and 0% slope; alignment mass window, 2 mDa intercept, and 20 ppm slope. The normalized features were filtered again, and only masses appearing at least in two of three samples were accepted. Background noise was removed by subtracting masses found in blank runs from filtered masses. The extracted ion chromatogram (EIC) of each standard or endogenous metabolite was extracted with ±20 ppm single ion expansion using MassHunter software v B.06.00. Statistical comparisons were conducted using a one-way ANOVA at the 0.05 level of significance and selecting a fold change > 2.0 to discriminate the differences between treated and control samples.

An in-house database comprising data from METLIN library and from the literature was employed to tentatively identify compounds using a mass accuracy of 10 ppm. Empirical formulas were generated for unknown compounds with the following parameters: ppm limit = 10, isotope model = common organic molecules, limit charge state to a maximum of 2, and use +H or −H, or sodium and potassium adducts. Standards confirmed the identification according to experimental migration time, UV max, and mass fragments.

## 3. Results

### 3.1. Effects on Disease Symptoms and Plant Growth

**Figure 1:**
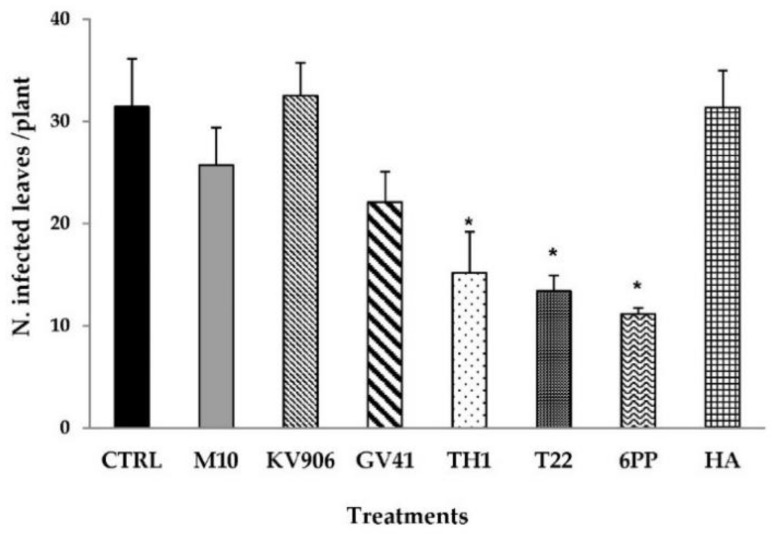
Number of olive leaves infected by *Fusicladium oleagineum* on plants treated with *Trichoderma* strains (M10, KV906, GV41, TH1, and T22) or secondary metabolites (6PP and HA). Plants treated with water (CTRL) were used as controls. Data are expressed as the mean value ± standard error. An asterisk (*) indicates values that are significantly different from the control (* *p* < 0.05; Student’s *t*-test).

During the field experiments carried out in this work, natural infections caused by the foliar pathogen *Fusicladium oleagineum* (syn. *Spilocaea aoleagina*) were observed on the olive trees cv. Carolea (susceptible to *F. olagineum*). Several treatments, both using *Trichoderma* metabolites and strains, determined a significant reduction of the leaf-spot symptoms caused by *F. olagineum* compared to water-treated plants (Figure 1). The mean number of infected leaves was found to be significantly decreased (*p* < 0.05) in plants treated with the SM 6-pentyl-α-pyrone (6PP: >60% reduction) or with *T. harzianum* strains T22 or TH1 (about 50% reduction), compared to the control (Figure 1). About 31 infected leaves were found in water-treated plants. Similar results were observed on olive trees treated with *Trichoderma* strain KV906 or the SM HA. No significant differences (*p* < 0.05) were observed in GV41- and M10-treated plants, as compared to the untreated control (Figure 1).

Field treatments also positively affected the growth and development of olive trees. After 6 months from the first application, the number of leaves and the branch length were particularly increased in T22-treated plants compared to the control (data not shown). Interestingly, the relative chlorophyll content in olive leaves also varied among treated plants. Chlorophyll content increased when plants were treated with strain KV906 or the metabolites 6PP and HA, as compared to other treatments, but no significant differences were observed vs. the control (Figure S1).

### 3.2. Induction of Plant Defense Responses

**Figure 2:**
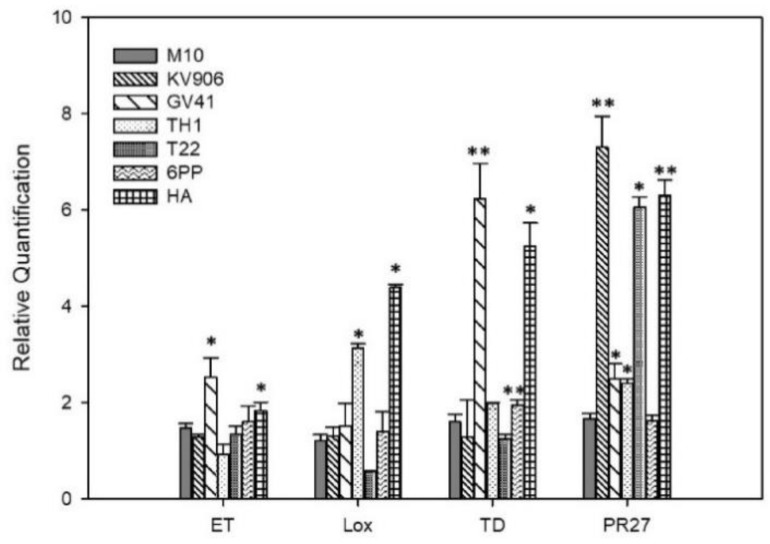
Relative quantification of defense-related transcripts on plants treated with *Trichoderma* strains (M10, KV906, GV41, TH1, and T22) or secondary metabolites (6PP and HA). Plants treated with water (CTRL) were used as background. Error bars refer to standard error. An asterisk (*) indicates values that are significantly different from the control (* *p* < 0.05; ** *p* < 0.01; Student’s *t*-test). ET: Ethylene-responsive transcription factor; Lox: lipoxygenase; TD: Tioredoxin; PR27: Pathogenesis-related protein 27.

The effect of *Trichoderma* treatments on the stimulation of olive defense responses was evaluated by monitoring the expression of a group of defense-related genes: (i) ethylene-responsive transcription factor (ET); (ii) lipoxygenase (LOX); (iii) tioredoxin (TD); and (iv) pathogenesis-related protein 27 (PR27). Figure 2 shows that experimental treatments positively influenced the expression of the genes under investigation. In particular, HA determined the upregulation of all the defense genes, while ET and LOX genes were induced also after the application of GV41 or TH1, respectively. Moreover, KV906 and T22 upregulated remarkably the PR27 gene, while TD expression was induced significantly (*p* < 0.001) by GV41.

### 3.3. Characterization of Olive Leaf Metabolome

**Figure 3:**
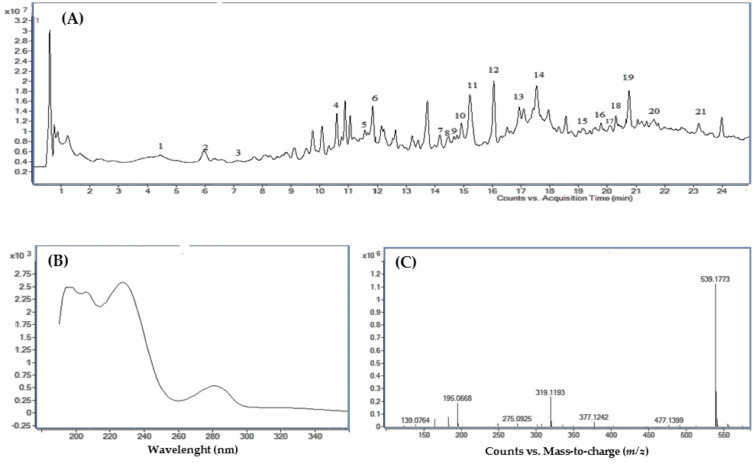
LC–MS Q-TOF analysis of an olive leaf extract. (**A**) Total ion chromatogram (TIC); (**B**) UV–vis, and (**C**) mass spectra of oleuropein (*m*/*z* = 539). Peak numbers in (**A**) are those reported in Table 1.

For each olive leaf sample, the total ion chromatogram (TIC), UV–VIS, and mass spectra (Figure 3) were analyzed and compared with the data reported in the literature. Table 1 reports the compounds putatively identified in olive leaf extracts including their retention time, experimental and calculated masses, molecular formula, UV maximum absorption, together with their proposed identities. LC–MS Q-TOF analysis showed the presence of 22 major compounds distinguished in five groups (secoiridoids, flavonoids, simple phenols, oleosides, and elenolic acids) according to their chromatographic and spectral characteristics (Table 1).

### 3.4. Untargeted Metabolomic Analysis of Leaf Extracts

An untargeted metabolomic approach was used to discriminate significant differences in the accumulation of phenolic compounds in leaves of olive plants treated with *Trichoderma* strains or metabolites. A total of 104 metabolites were detected from olive leaf extracts, of which about 20 were putatively identified using the METLINE library and an in-house database containing more than 200 plant secondary metabolites. Statistical analysis by one-way ANOVA revealed the presence of 88 compounds that accumulated differently (*p* < 0.05) in the leaf extracts of treated plants vs. controls. Among these, 23 showed a consistent variation (fold change ≥ 2.0; Table 2). However, the highest number of metabolites differentially accumulated compared to the control were those whose relative abundance decreased after the field application of M10, HA, or 6PP (Table 2).

**Figure 4:**
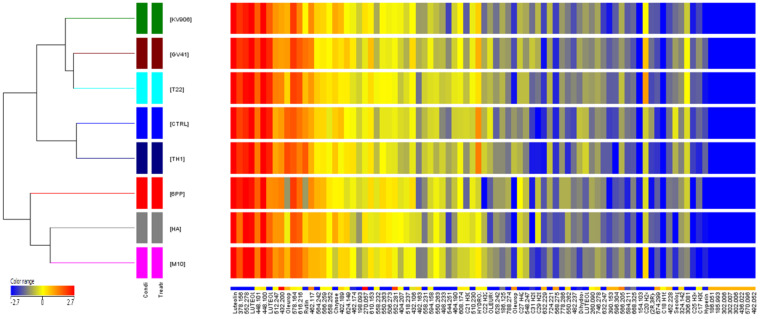
Hierarchical clustering heat map of differential metabolic profiles from olive leaf extracts. Samples are indicated according to the treatments with *Trichoderma* strains (M10, KV906, GV41, TH1, and T22) or secondary metabolites (6PP and HA). Plants treated with water (CTRL) were used as controls. Red and blue colors indicate, respectively, a relatively higher and lower chemical abundance. Yellow indicates a neutral change from the overall average abundance found across all samples. Each column represents one metabolite, with rows on the left having an overall higher level of perturbation across the metabolomes. The heat map was developed by using Agilent MassProfiler Professional bioinformatics software and statistical differences were determined through ANOVA statistical testing (one-way ANOVA, *p* < 0.05).

A hierarchical cluster analysis was carried out by grouping replicate samples based on the abundance of continuous variables (one-way ANOVA, *p* < 0.05). The dendrogram shown in Figure 4 was obtained analyzing the 88 metabolites differentially accumulated among treatments and comparing their chemical abundance vs. control (water-treated plants). The metabolic profiling revealed a different distribution of compounds in treated plants compared to control; in particular, leaf samples from olive plants treated with the *Trichoderma* strain M10 or the SMs 6PP or HA (produced by strain M10) grouped totally separately from the others. Another group was constituted by samples of plants treated with *Trichoderma* strains T22, GV41, or KV906. On the other hand, the application of strain TH1 determined no differences in terms of accumulation of phenolic compounds compared to the control (Figure 4). Overall, we found that among treated plants, the majority of the 88 differential metabolites showed a lower chemical abundance (blue color), while in the control a lower number of differential compounds with lower chemical abundance was observed.

**Figure 5:**
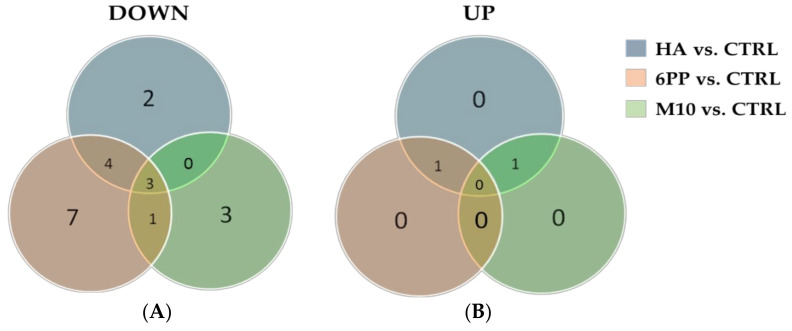
Venn diagrams of phenolic compounds whose abundance in the olive leaf metabolome (**A**) decreased (DOWN) or (**B**) increased (UP) compared to the control (CTRL). Data refer to metabolites extracted from plants treated with *Trichoderma* metabolites (HA and 6PP) or *T. harzianum* strain M10.

Thus, considering the treatments that showed the greatest differences in terms of metabolite accumulation compared to control (6PP, HA, or M10; Figure 4), we further analyzed the variations in metabolite accumulation among increased (UP) or decreased (DOWN) compounds, as compared to the control (Figure 5). Venn diagram of metabolites among DOWN groups showed that three compounds were in common to these groups, while seven metabolites were those exclusively present in the metabolome of 6PP-treated samples, three in those exposed to M10, and only two in HA-treated plants. These included several compounds belonging to different phenolic groups, such as oleuropein, luteolin, rutin, luteolin rutinoside, chrysoeriol, and hydroxytyrosol-hexose (Table S1). Interestingly, four compounds showed a decreased accumulation vs. control in both plants treated with the *Trichoderma* metabolites 6PP or HA, but none were in common between M10- and HA-treated plants. Conversely, less numerous were the differential metabolites whose intensity increased compared to water-treated plants when exposed to 6PP, HA, or M10 (Figure 3, Table S1).

### 3.5. Targeted Metabolomic Analysis of Leaf Extracts

In this work, the phenolic compounds were quantified according to their group: the calibration curve of oleuropein was used to quantify oleuropein and other secoiridoids; the calibration curves of luteolin and apigenin were used to quantify flavonoids; the calibration curve of hydroxytyrosol was used to quantify simple phenols and elenolic acids. The calibration curves revealed good correlation between peak areas and analyte concentrations, and the regression coefficients were 0.99, with the only exception of commercial apigenin (r^2^ = 0.96; Table S3).

Phenolic contents are reported in Table 3 as mg of compound g^−1^ dry olive leaves. We found that hydroxytyrosol-hexose, and derivatives of oleuropein (isomer a, b, and diglucoside) and luteolin (rutinoside and diglucoside) were the most abundant compounds in all the olive leaf extracts (Table 3). However, significant differences in the accumulation of phenolic compounds in olive leaves were determined by the plant treatment (Table 3).

Among the secoiridoids, numerous compounds, including oleuropein isomers and its diglucoside, were less abundant in treated plants as compared to the control; conversely, the accumulation of oleuropein aglycon increased up to 42% in olive plants subjected to TH1 or 6PP (Table 3).

The analysis of leaf extracts revealed a general increase of flavonoids in treated plants vs. controls. This was found to be statistically significant for luteolin derivatives (i.e., luteolin rutinoside and luteolin diglucoside), and for chrysoeriol-7-O-glucoside, apigenin rutinoside, and apigenin glucoside, in plants treated with *Trichoderma* strains or metabolites (Table 3). In some cases, the treated plants showed a decreased accumulation in the content of specific flavonoids (i.e., luteolin and rutin). The contents of the simple phenol hydroxytyrosol-hexose or the oleoside secologanoside decreased when olive plants were treated with the *Trichoderma* strain M10 or KV906, and with its SMs 6PP or HA (Table 3). On the other hand, the concentration of elenolic acid glucoside increased about 67% in HA-treated plants compared to the control.

### 3.6. Effect of Treatments on the Expression of Genes Involved in Oleuropein Biosynthesis

**Figure 6:**
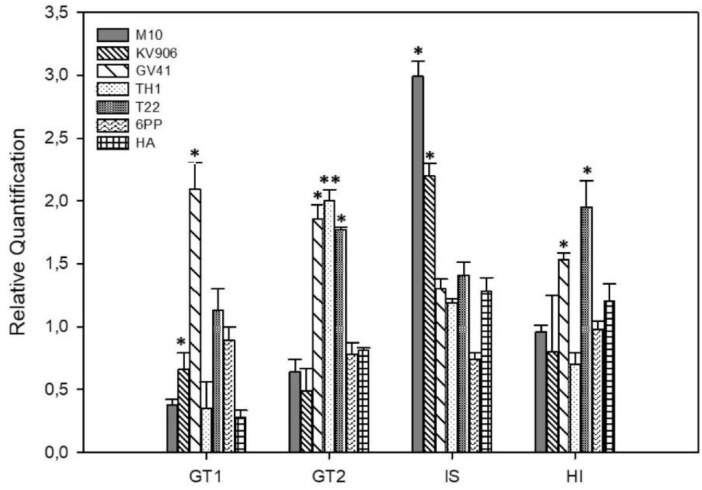
Relative quantification of genes active in the oleuropein biosynthetic pathway on plants treated with *Trichoderma* strains (M10, KV906, GV41, TH1, and T22) or secondary metabolites (6PP and HA). Plants treated with water (CTRL) were used as a calibrator. Error bars refer to standard error. An asterisk (*) indicates values that are significantly different from the control (* *p* < 0.05; ** *p*< 0.01; Student’s *t*-test). GT1 and GT2: glucosyl transferase 1 and 2, respectively; IS: Iridoid synthase; HI: hydroxylase.

Figure 6 shows the expression of four genes involved in oleuropein biosynthesis in olive leaves collected from treated plants Genes under investigation were: an iridoid synthase (IS), coding for a central enzyme catalyzing the production of an essential oleuropein intermediate; glucosyl transferase 1 and 2 (GT1 and GT2, respectively), genes coding for enzymes acting downstream loganin production; and hydroxylase (HI), which catalyzes the final reaction of ligsostride conversion to oleuropein (Figure S2). The expression of these genes was differently affected by the experimental treatments. Strain GV41 determined a significant (*p* < 0.05) up-regulation of GT1, GT2 and HI, while strain M10 increased the expression of IS. Significant upregulation of the selected genes was also observed following the application of strains TH1 or T22 in the case of GT2, strain KV906 for GT1 and IS, and strain T22 for HI (Figure 6). Interestingly, neither of the SMs applied (HA or 6PP) influenced the expression of the genes active in the biosynthesis of oleuropein.

## 4. Discussion

In the present study the effects of *Trichoderma* strains or metabolites on the growth of young olive trees, and their influence on the development of natural infections caused by *F. oleagineum*, were investigated. Fungal isolates were selected among strains that showed antagonistic and PGP activities previously tested in our lab. Similarly, the *Trichoderma* SMs HA and 6PP, already isolated and characterized as antibiotics, plant resistance inducers, and root stimulators [37,39], were used in the present study. Our results showed that strains TH1 and T22 (the latter already marketed as MBCA in numerous commercial formulations) and the metabolite 6PP determined a significant reduction of foliar symptoms associated to the olive peacock spot disease. These consisted of brown, yellow, or green circular spots whose circumference increased over time from a few millimeters to occupy the whole leaf. The development of this disease has been correlated to climate conditions; whereby optimal fungal conidia germination occurs when relative humidity is very high and temperature ranges between 10 and 20 °C [52]. Our results suggest that *Trichoderma* fungi may act as MBCAs to contrast foliar pathogens of olive trees, as previously reported also for soil borne disease agents [53,54]. Recent studies investigated the ability of *T. asperellum* strain T25 to colonize olive rhizosphere and reduce the severity of symptoms caused by *Verticillium dahliae* in controlled conditions [43,55] Moreover, a growth promoting effect was observed in T25-inoculated olive plants also under severe Verticillium wilt infection [43]. We also observed a growth promoting effect of *Trichoderma* fungi or metabolites: six months after the first treatment a considerable increase in leaf number and branch length was registered as being associated to the application of strains T22 and KV906, or the SMs 6PP (data not shown). These data agree with previous observations where different *Trichoderma* strains or metabolites were applied on different crops [56,57,58,59,60,61,62].

Considering that *Trichoderma* is among the most important fungal genera able to effectively control various plant pests, both pathogens and insect herbivores directly and indirectly (including the induction of endogenous plant defense) [33,35,63], we evaluated the effects of repeated applications of *Trichoderma* spores or SMs on the induction of olive defense responses. The expression of four defense genes associated with different hormonal pathways involved in plant defense (jasmonic acid, salicylic acid, and ethylene) was investigated. The selected genes represent some of the major mediators of plant defense responses to biotic stresses and environmental threats [64,65,66,67], and included: (i) ET, which encodes for a transcription factor belonging to the family APETALA2/ERF involved in ethylene signaling and the response pathway, in particular in hormonal cross-talk [68,69]; (ii) LOX, encoding for an enzyme that catalyzes the early steps of jasmonic acid (JA) biosynthesis, and influence local and systemic responses against many biotic and abiotic stresses [70]; (iii) TD, encoding for an ubiquitous disulfide reductase, enzymes that regulate the redox status of target proteins and play an important role in reactive oxygen species (ROS) scavenging during stress responses [71]; and (iv) PR27, encoding for a PR-protein included in a group of plant proteins induced in response to fungal, bacterial, viral, and viroid infection, and to some chemicals [65,66]. Interestingly, the fungal metabolite HA caused the upregulation of all selected defense genes, while the strains GV41 and KV906 produced a differential modulation of gene expression. The upregulation of TD by several tested treatments was consistent with the induction of a ROS-mediated response observed in the olive response to the bacterial pathogen *Xylella fastidiosa* [72]. Plants purposefully generate ROS as signaling molecules to control various processes including defense to pathogen attack and programmed cell death [73], thus the instauration of the ROS signaling in the treated olive trees could represent the link to the observed activation of multiple defensive pathways, controlled by different plant hormones. These results may explain, at least in part, the reduction of disease symptoms observed in treated olive trees and suggest a possible protection against various pests. In addition, since *Trichoderma* may act also by inducing systemic resistance and promoting plant growth [34,35,62], it is likely that it may facilitate the plant to counteract the detrimental effects of pathogens, as observed in this work.

Numerous studies carried out so far on the phenolic compounds in *O. europaea* focused on olive fruits or oil, probably due to their economic importance in the food industry [17,18,74,75,76]. Similarly, a few studies investigated the involvement of olive phenols in the defense against the fungal pathogens *F. oleagineum* and *V. dahliae* [20,21,27,28], these being the compounds often associated with plant host resistance [28,77]. In our study, the production of phenolic compounds in olive leaves has been evaluated in relation to the application of biological treatments in the field. Olive leaves are considered as a valuable waste byproduct of the oil industry and may represent a source of bioactive compounds since they have a long history of medicinal value [7,78]. The untargeted analysis of olive leaf metabolome allowed the evaluation of significant differences between plants treated with the different *Trichoderma* strains or metabolites. This approach has been used in other studies on different plants and with different techniques [79,80]. Previous studies on olive leaves revealed differences in individual and total phenolic content depending upon both the season and the cultivar [18]. Here, the analysis of chromatographic phenolic profiles revealed about 20 compounds that accumulated differently among the leaf extracts. After 6 months of application of *Trichoderma* spores or metabolites to olive plants, phenolic compounds generally decreased in treated samples compared to controls. A clear separation was observed in the metabolomic profiles of olive leaves following the application of the SMs HA and 6PP, or the fungal strain M10, in comparison to all other treatments. This result may indicate that the plant responds similarly when inoculated with the living microbe or its bioactive compound [37,42,62]. However, the identified differential metabolites were not always common to all three treatments, as shown in Figure 5.

If untargeted studies provide a comprehensive evaluation of metabolomics profiles, targeted studies focus on the analysis of a specific set of known metabolites [81]. Here a targeted metabolomic approach was used to evaluate the accumulation of phenolic compounds in olive leaves and the differences were evaluated relative to the plant treatment and the class of compounds (secoiridoids, flavonoids, simple phenols, oleosides, and elenolic acids glucosides). Among secoiridoids a decrease in the content of oleuropein and its glucosidate adduct was observed in most of the samples treated with *Trichoderma* vs. control, which corresponded to an increase in derivatives (aglycone and methylated). This can be related to the degradation of oleuropein and its transformation into derivatives, normally noted in autumn [82], and that field treatments may have accelerated. In addition, the upregulation of genes involved in oleuropein biosynthesis following application of *Trichoderma* strains (i.e., GV41 or M10) may have also supported such a transformation. Very recently, secoiridoids and oleuropein were determined to have a more important role in *O. europaea* protection against UV-B radiation by acting as signaling molecules as part of the antioxidant defense machinery [83]. Thus, the induction of genes involved in the synthesis and modification of oleuropein was perhaps not an isolated phenomenon, but could be linked to the upregulation of genes involved in ROS signaling and other defense-related genes suggesting a more complex and tangled regulation of gene expression upon our treatments.

Conversely, the flavonoids apigenin rutinoside and crisoeriol 7-0-glucoside showed a marked increase in the accumulation after all *Trichoderma* treatments compared to the control. Finally, for the elenolic acid, the treatments that demonstrated an increase in the accumulation of this compound were those performed with the application of 6PP and HA metabolites and with KV906 strain. These results, and in particular those obtained with the fungal metabolites, are in agreement with the study conducted by Pascale et al. [62], where an increase in total polyphenol content is reported in the grapes treated with the spores of the *T. harzianum* strain T22 or with 6PP.

## 5. Conclusions

The application of beneficial microorganisms of the genus *Trichoderma* or their bioactive metabolites influenced the growth and development of young olive plants (*O. europaea* cv. Carolea). The treatments determined an increase in plant biometric parameters (number of leaves and length of branches), and a reduction in the number of leaves affected by peacock spot disease compared to controls. This latter phenotype appears to be associated with the increased expression of defense-related genes that may contribute to the observed reduction of disease symptoms. The LC–MS analysis of olive leaf metabolome also revealed a variation in the content of phenolic compounds, frequently associated to plant host resistance. To our knowledge, this is the first report demonstrating the effects exerted by *Trichoderma* strains or metabolites on olive leaf metabolome resulting also in the activation of defense-related genes. This evidence offer new opportunities for the development of bioformulations based on microorganisms or their metabolites to be used also on tree crops, such as olive. In the future, it will be important to assess whether the effects of these field treatments conducted with *Trichoderma* and/or their bioactive compounds in relation to the content of polyphenols in young olive plants can also be observed in adult olive trees and in drupes.

## Figures and Tables

**Table 1:** List of phenolic compounds putatively identified in the extract of olive leaves from treated plants by LC–MS Q-TOF. Data include compound number (N.) as indicated in Figure 2, retention time (RT, min), phenolic group, experimental and theoretical mass, calculated molecular formula, and UV maximum absorption (nm).

N.	RT (min)	Compound	Group *	Formula	Experimental Mass	Theoretical Mass	UV Max (nm)
1	4.75	Hydroxytyrosol-hexose	3	C_14_H_20_O_8_	316.1160	315.10	230; 280
2	6.33	Oleoside methyl ester	4	C_17_H_24_O_11_	404.1321	404.36	235
3	7.45	Secologanoside	4	C_16_H_22_O_11_	390.1151	390.34	234
4	10.90	Oleuropeinaglycon	1	C_16_H_26_01_6_	378.1569	378.38	235; 271
5	11.84	Elenolic acid glucoside	5	C_17_H_24_0_11_	404.1325	404.13	248; 274
6	11.90	Luteolin rutinoside is. a	2	C_27_H_30_0_15_	594.1589	594.52	248; 267
7	12.25	Luteolin di-glucoside	2	C_27_H_30_0_16_	610.1537	610.15	248; 267; 335
8	14.55	Verbascoside	3	C_29_H_36_O_15_	624.2064	624.59	234; 329
9	14.60	Rutin	2	C_27_H_30_0_16_	610.1539	610.52	253
10	14.90	Luteolin rutinoside is. b	2	C_27_H_30_0_15_	594.1592	594.52	253; 347
11	15.18	Chrysoeriol-7-O-glucoside	2	C_22_H_22_O_11_	462.1497	462.40	250; 347
12	15.81	2-Methoxyoleuropein	1	C_26_H_34_O_14_	570.1942	570.17	236; 280
13	16.90	Apigeninrutinoside	2	C_27_H_30_0_14_	578.1640	578.16	237; 266
14	17.40	Apigenin glucoside	2	C_21_H_20_O_10_	432.1061	432.38	345
15	17.90	Oleuropeindiglucoside	1	C_31_H_24_O_18_	702.2400	702.66	235; 277
16	19.10	Oleuropein isomer a	1	C_25_H_32_O_13_	540.1840	540.18	240; 280
17	19.89	Oleuroside	1	C_25_H_32_O_13_	540.1845	540.51	230; 280
18	20.10	Oleuropein isomer b	1	C_25_H_32_O_13_	540.1848	540.18	235; 280
19	20.52	Ligstroside	1	C_25_H_32_0_12_	524.1900	524.52	230; 280
20	20.93	Apigenin	2	C_15_H_10_O_5_	270.0530	270.24	268; 334
21	20.80	Luteolin	2	C_15_H_35_O_14_	286.0488	286.05	255; 286
22	23.28	Chrysoeriol	2	C_16_H_12_O_6_	300.0682	300.26	198

* 1, secoiridoids; 2, flavonoids; 3, simple phenols; 4, oleosides; 5, elenolic acids glucosides [18].

**Table 2:** Number of metabolites found in olive leaf extracts whose accumulation increased (UP) or decreased (DOWN) compared to the control (CTRL) after 5 field applications with *Trichoderma* strains or metabolites. Data were analyzed by one-way ANOVA using Mass Profiler software (Agilent Technologies) and only statistically significant compounds (*p* < 0.05) that accumulated in the metabolome of treated plants vs. control (fold change ≥ 2.0) have been reported.

*Trichoderma*	Treatment	UP vs. CTRL	DOWN vs. CTRL
Strain	GV41	3	1
	M10	1	7
	T22	2	3
	TH1	0	0
	KV906	1	5
Metabolite	HA	1	9
	6PP	2	15

**Table 3:** Phenolic compounds detected in leaf extracts of olive plants subjected to field applications of *Trichoderma* strains (M10, KV906, GV41, TH1, and T22) or secondary metabolites (HA and 6PP). CTRL = samples from water-treated plants. Values are expressed as mg g^−1^ of dry olive leaves and standard deviation (in parenthesis). Different letters in a row indicate statistically significant differences (one-way ANOVA at the 0.05 level of significance).

			TREATMENT							
RT (min)	Compound	Mass	CTRL	M10	KV906	GV41	TH1	T22	6PP	HA
4.75	Hydroxytyrosol-hexose	316	0.947 (0.700) ^b,c^	0.448 (0.198) ^a^	0.391 (0.087) ^a^	0.515 (0.265) ^a,b^	1.203 (0.833) ^c^	0.350 (0.146) ^b,c^	0.241 (0.162) ^a^	0.341 (0.130) ^a^
7.45	Secologanoside	390	0.320 (0.020) ^b^	0.280 (0.040) ^a^	0.152 (0.024) ^a^	0.240 (0.072) ^a^	0.418 (0.022) ^b^	0.160 (0.022) ^a^	0.160 (0.040) ^a^	0.080 (0.064) ^a^
10.90	Oleuropein glycon	378	0.038 (0.001) ^a,c^	0.045 (0.002) ^b,c,d,e^	0.041 (0.002) ^a,d^	0.035 (0.001) ^a^	0.054 (0.010) ^e^	0.036 (0.001) ^a,b^	0.051 (0.004) ^d,e^	0.032 (0.001) ^a^
11.84	Elenolic acid glucoside	404	0.206 (0.058) ^a,b^	0.174 (0.003) ^a,b^	0.214 (0.002) ^a,b^	0.137 (0.003) ^a,b^	0.167 (0.004) ^a,b^	0.125 (0.002) ^a^	0.227 (0.095) ^b^	0.344 (0.048) ^c^
11.90	Luteolin rutinoside is. a	594	0.312 (0.160) ^a,b^	0.360 (0.032) ^c^	0.240 (0.016) ^a^	0.320 (0.016) ^a,b^	0.344 (0.032) ^b,c^	0.280 (0.005) ^a^	0.280 (0.024) ^a^	0.304 (0.040) ^a,b^
12.25	Luteolin diglucoside	610	0.384 (0.040) ^a,b^	0.600 (0.056) ^d^	0.376 (0.016) ^a^	0.440 (0.048) ^c^	0.464 (0.064) ^c,d^	0.392 (0.024) ^a,b^	0.416 (0.072) ^b,c^	0.360 (0.016) ^a^
14.60	Rutin	610	0.211 (1.192) ^c,d^	0.119 (0.040) ^b,c,d^	0.048 (0.036) ^a,b^	0.077 (0.038) ^b,c,d^	0.257 (0.102) ^d^	0.050 (0.035) ^a,b^	0.016 (0.020) ^a^	0.068 (0.040) ^a,c^
14.90	Luteolin rutinosideis. b	594	0.329 (0.026) ^c,d^	0.313 (0.016) ^c,d^	0.139 (0.011) ^a,b^	0.231 (0.021) ^b,c^	0.411 (0.037) ^d^	0.253 (0.018) ^b,c^	0.047 (0.003) ^a^	0.177 (0.014) ^a,b^
15.18	Chrysoeriol-7-O-glucoside	462	0.007 (0.001) ^a^	0.035 (0.002) ^a,b^	0.047 (0.004) ^b^	0.036 (0.003) ^a,b^	0.024 (0.002) ^a,b^	0.014 (0.001) ^a,b^	0.045 (0.003) ^b^	0.027 (0.002) ^a,b^
15.81	2-Methoyxyoleuropein	570	0.400 (0.032) ^a,c^	0.160 (0.008) ^b,c^	0.152 (0.012) ^b,c^	0.500 (0.045) ^d^	0.280 (0.025) ^a,c^	0.400 (0.028) ^a,c^	0.080 (0.005) ^a,b^	0.064 (0.005) ^a^
16.90	Apigenin rutinoside	578	0.382 (0.040) ^a^	0.574 (0.040) ^b^	0.517 (0.034) ^a,b^	0.447 (0.037) ^a,b^	0.492 (0.038) ^a,b^	0.439 (0.020) ^a,b^	0.557 (0.060) ^b^	0.563 (0.044) ^b^
17.40	Apigenin glucoside	432	0.064 (0.004) ^a^	0.088 (0.006) ^c^	0.080 (0.007) ^a,b^	0.084 (0.006) ^b,c^	0.072 (0.004) ^a,b^	0.068 (0.005) ^a,b^	0.096 (0.012) ^c^	0.064 (0.006) ^a^
17.90	Oleuropein diglucoside	702	0.680 (0.320) ^c^	0.520 (0.280) ^b^	0.480 (0.056) ^a,b^	0.536 (0.048) ^b,c^	0.600 (0.320) ^b,c^	0.440 (0.048) ^a,b^	0.400 (0.060) ^a^	0.416 (0.056) ^a^
19.10	Oleuropein isomer a	540	8.428 (0.849) ^b,c^	2.106 (0.068) ^a^	2.192 (0.169) ^a,b^	1.608 (0.044) ^a^	11.694 (1.171) ^c^	0.933 (0.023) ^a^	0.480 (0.036) ^a^	0.646 (0.052) ^a^
20.10	Oleuropein isomer b	540	1.062 (0.085) ^b^	0.144 (0.007) ^a^	0.160 (0.013) ^a^	0.120 (0.011) ^a^	1.661 (0.150) ^b^	0.080 (0.006) ^a^	0.048 (0.003) ^a^	0.080 (0.006) ^a^
20.80	Luteolin	286	0.129 (0.049) ^c^	0.088 (0.031) ^a,c^	0.086 (0.023) ^a,b,c^	0.068 (0.004) ^a,b^	0.119 (0.042) ^c^	0.101 (0.021) ^b,c^	0.049 (0.002) ^a^	0.119 (0.003) ^c^
23.28	Chrysoeriol	300	0.053 (0.004) ^a,b,c^	0.036 (0.002) ^a^	0.084 (0.007) ^b,c,d^	0.040 (0.004) ^a,b^	0.096 (0.009) ^c,d^	0.048 (0.003) ^a,b^	0.038 (0.002) ^a,b^	0.104 (0.008) ^d^

## Supplementary Materials

The following are available online at https://www.mdpi.com/2309-608X/6/4/369/s1, Figure S1: Chlorophyll content in olive leaves of plants treated with *Trichoderma* strains (M10, KV906, GV41, TH1, and T22) or secondary metabolites (6PP and HA). Figure S2: Schematic and simplified representation of olive secoiridoid pathway. Table S1: Comparison of metabolites in different olive leaf metabolomes whose accumulation increased (UP) or decreased (DOWN) compared to control (CTRL) after 5 field applications with *Trichoderma* strains or metabolites. Table S2: List of primers used in this study. Table S3: Analytical parameters of the commercial standards used for the quantification of phenolic compounds in olive leaf extracts.
