# Supplementary material for: The Application of Trichoderma Strains or Metabolites Alters the Olive Leaf Metabolome and the Expression of Defense-Related Genes
Source: J Fungi (Basel). 2020 Dec 16;6(4):369. doi: 10.3390/jof6040369 (PMC7766153; doi:10.3390/jof6040369)
Supplement: Supplementary file 1 [file jof-06-00369-s001.pdf]

Journal of Fungi

MDPI

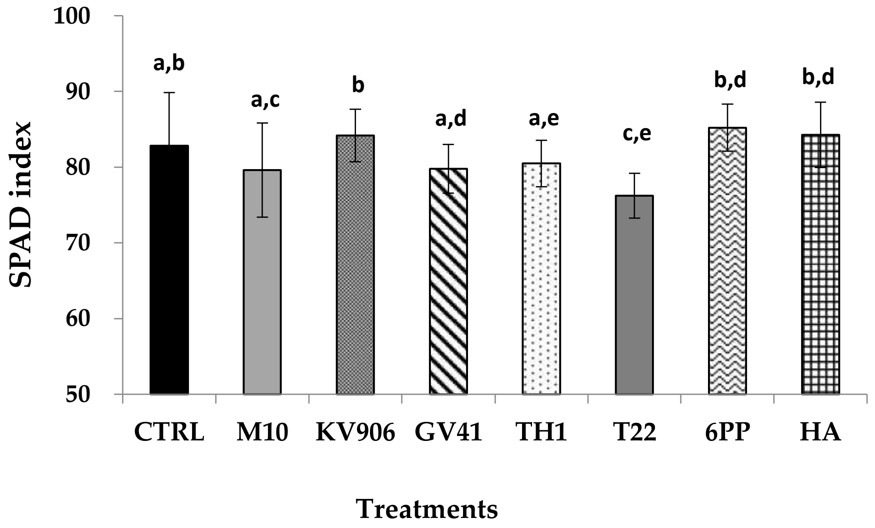

Figure S1. Chlorophyll content in olive leaves of plants treated with *Trichoderma* strains (M10, KV906, GV41, TH1, T22) or secondary metabolites (6PP, HA). Plants treated with water (CTRL) were used as controls. Different letters indicate statistically significant differences for $p < 0.05$, as analyzed by one-way ANOVA.

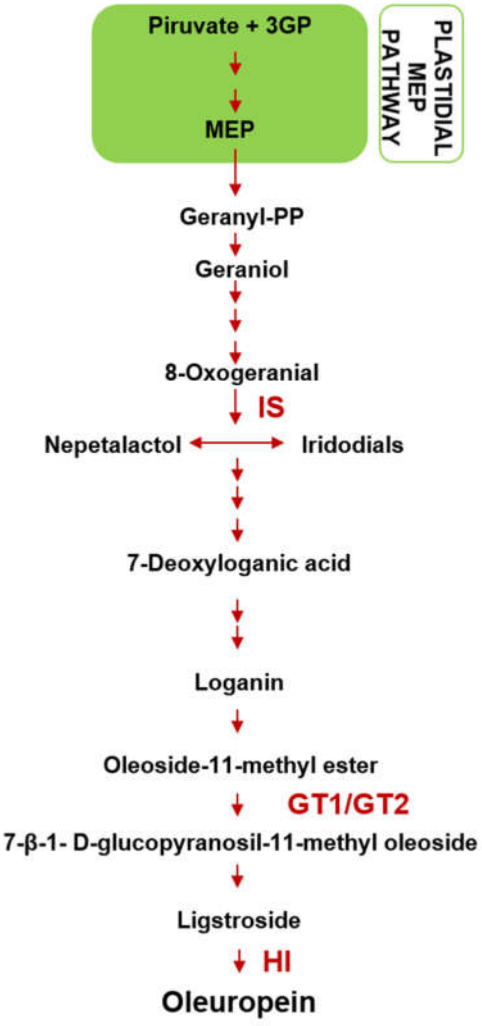

Modified from Alagna et al. J. Biol. Chem. 2016;291:5542-5554

Figure S2. Schematic and simplified representation of olive secoiridoid pathway. Iridoid synthase (IS) coding for a central enzyme catalyzing the production of a central oleuropein intermediate; glucosyl transferase 1 and 2 (GT1 and GT2, respectively), genes coding for enzymes acting downstream loganin production; hydroxylase (HI) which catalyses the final reaction of ligsostride conversion to oleuropein.

Table S1. Comparison of metabolites in different olive leaf metabolomes whose accumulation increased (UP) or decreased (DOWN) compared to control (CTRL) after 5 field applications with *Trichoderma* strains or metabolites. Only statistically significant compounds (p< 0,05) that accumulated in the metabolome of treated plants vs. control (fold change ≥ 2.0) have been reported. The phenolic compounds identified by metabolomic profiling have been reported as indicated in **Table 1** (in parenthesis), while unmatched compounds are indicated using the molecular formula proposed by Mass Profiler software (Agilent Technologies) including their mass and retention time (in parenthesis).

**Both in 6PP and HA (tot 4)**	**Both in M10 and HA (tot 0)**	**Both in M10 and 6PP (tot 1)**	**Both in 6PP, M10 and HA (tot 3)**	**Only in 6PP (tot 7)**	**Only in M10 (tot 3)**	**Only in HA (tot 2)**
Luteolin glucoside (448/15.2)	-	$C_{15}H_{12}O_6$ (288/20.0)	Hydroxytyrosol-hexose (1)	Luteolin (20)	Chrysoeriol (21)	Luteolin rutinoside (594/15.8)
Oleuropein is. a (15)			Oleuropein is. b (17)	Luteolin rutinoside (9)	Dihydroquercetin (304/13.7)	$C_{31}H_{28}N_3O_8$ (570/18.7)
Oleuropein diglucoside (14)			Secologanoside (3)	Rutin (8)	$C_{17}H_{30}N_7$ (332/24.6)	
$C_{18}H_{16}N_7O_3$ (378/19.0)				$C_{21}H_{32}O_{13}$ (490/9.5)		
				$C_{22}H_{32}O_{14}$ (520/10.6)		
				$C_{25}H_{34}O_{12}$ (526/19.9)		
				$C_{21}H_{20}O_{12}$ (464/15.0)		

**In common to 6PP & HA (tot 1)**	**In common to M10 & HA (tot 0)**	**In common to M10 & 6PP (tot 1)**	**In common to all (tot 0)**	**Only in 6PP (tot 0)**	**Only in M10 (tot 0)**	**Only in HA (tot 0)**
$C_{29}H_{28}O_{14}$ (600/20.5)	-	$C_{27}H_{42}O_{14}$ (590/17.1)	-	-	-	-

Table S2. List of primers used in this study.

Primer sequence (5'-3')	Gene	Abbreviation
ATCCAAACGCCAAAAATCAG	Iridoid synthase	IS
ATCCCACCATCTCCAAGTCA		
AAAAACACAACGGCACCACT	Olive glucosyl transferase 1	GT1
TTCTCTTCGGCAAAATCACC		
CAGGCTTCCAGGCTATCAAA	Olive glucosyl transferase 2	GT2
GGGTCCTTCCAAATCTTCAA		
TGGAAGGAAGTCTGGAATGAG	Hydroxilase	HI
CACAAGCAAGGATGATGTCG		
CGGGTTGGACCAGTGAATGT	Lipoxigenase	LOX
TTGACACACTGTTGGGAATTCC		
CCCAGAAGATCAACGAAGTAGGTG	Pathogenesis-related protein 27	PR27
GACCGCACGATTCTTGGATT		
TCATGTGCGTTGTTGATGGT	Ethylene-responsive Transcription Factor	ET
TGTCTTTTAACATTCACACAGA		
TAGCACTGGCACTGAGGAGGATT	Tioredoxin	TD
TCTCGAGTTGTGACATGCTT		
CCTCTTGGACGATTTGCTGT	Elongation factor 1-α	EF1α
CCTGTTGGCTCCTTCTTGTC		

Table S3. Analytical parameters of the commercial standards used for the quantification of phenolic compounds in olive leaf extracts.

Standard	Calibration Range [µg/ml]	Calibration equations	r^2
**Oleuropein**	0,625-25	y = 3264818,79x + 8921367,34	0,99
**Luteolin**	0,5-25	y = 2750905,36x + 2774612,53	0,99
**Apigenin**	0,5-50	y= 1388397,18x + 7259647,69	0,96
**Hydroxytyrosol**	0,62-62,5	y = 858369x + 1000000	0,99
